# Nonalcoholic steatohepatitis-associated hepatocarcinogenesis in mice fed a modified choline-deficient, methionine-lowered, L-amino acid-defined diet and the role of signal changes

**DOI:** 10.1371/journal.pone.0287657

**Published:** 2023-08-03

**Authors:** Noriko Suzuki-Kemuriyama, Akari Abe, Sae Nakane, Megumi Yuki, Katsuhiro Miyajima, Dai Nakae

**Affiliations:** 1 Faculty of Applied Bioscience, Department of Nutritional Science and Food Safety, Tokyo University of Agriculture, Setagaya, Tokyo, Japan; 2 Department of Nutritional Science and Food Safety, Graduate School of Applied Bioscience, Tokyo University of Agriculture, Setagaya, Tokyo, Japan; 3 Faculty of Health Care and Medical Sports, Department of Medical Sports, Teikyo Heisei University, Ichihara, Chiba, Japan; The Second Xiangya Hospital of Central South University, CHINA

## Abstract

Nonalcoholic steatohepatitis (NASH) can progress to cirrhosis and even hepatocellular carcinoma (HCC). The incidence of NASH-associated HCC is increasing, posing a serious public health threat. Unfortunately, the underlying pathological mechanisms, including the possible differences between neoplastic and non-neoplastic lesions, remain largely unknown. Previously, we reported a dietary mouse NASH model with a choline-deficient, methionine-lowered, L-amino-acid-defined, high-fat diet containing shortening without *trans* fatty acids (CDAA-HF-T[−]), which rapidly induces fibrosis and proliferative lesions in the liver. This study aimed to develop a mouse CDAA-HF-T(−) model capable of assessing NASH-associated hepatocarcinogenesis and identifying key signaling factors involved in its underlying mechanisms. Multiple large masses, histopathologically hepatocellular adenomas and carcinomas, and hemangiosarcomas were detected in the liver samples of mice fed CDAA-HF-T(−) for 52 or 63 weeks, along with highly advanced fibrosis and numerous foamy, phagocytic macrophages in the adjacent nontumoral area. Multiple metastatic nodules were found in the lungs of one of the animals, and lymphoid clusters were found in all CDAA-HF-T(−) group mice. In the Ingenuity Pathways Analysis of RNA expression data, the CDAA-HF-T(−) feeding revealed common signal changes in nontumoral and tumoral liver tissues, including increased IL-8 and RhoGTPases signaling and decreased lipid metabolism. Meanwhile, *macrophage inflammatory protein 2* (*MIP-2*) expression levels were upregulated in nontumoral liver tissue from the end of Week 13 of CDAA-HF-T(−) feeding to the end of Week 63. On the other hand, MIP-2 was expressed on macrophages in non-tumor areas and hepatocytes in tumor areas. Therefore, the CDAA-HF-T(−) mouse model is useful for assessing NASH and NASH-associated hepatocarcinogenesis, and IL-8 signaling plays important roles in NASH-associated carcinogenesis and cirrhosis, but it may also play different roles in nontumoral liver tissue and tumorigenesis.

## Introduction

Globally, liver cancer is one of the most common primary malignancies and a leading cause of cancer-related deaths [1]. Hepatocellular carcinoma (HCC) in particular accounts for 80%–90% of all primary liver cancers in adults [2]. Hepatitis B and C virus infections have long been recognized as major risk factors for HCC, but nonalcoholic fatty liver disease (NAFLD) has recently emerged as one of its leading causes [3]. NAFLD encompasses a wide range of liver diseases, the most severe of which is nonalcoholic steatohepatitis (NASH). NASH is characterized by steatosis, inflammation, ballooning, and the subsequent death of hepatocytes, resulting in hepatic fibrosis and cirrhosis, and is frequently associated with cancer [4–6]. The incidence of NASH-associated HCC (NASH-HCC) is rising, posing a serious public health threat [7]. However, the pathological mechanisms underlying NASH-associated hepatocarcinogenesis, including any differences between neoplastic and non-neoplastic lesions, remain unknown.

Researchers have attempted to elucidate the pathological mechanisms of NASH-HCC using several genetically modified mouse models [8], such as farnesoid X receptor knockout mice [9], melanocortin 4 receptor (Mc4r) knockout mice [10] and transmembrane 6 superfamily member 2 (TM6SF2) knockout mice and rats [11–13] as well as non-genetically modified animal models that receive mutagenic reagents (e.g., streptozotocin) [14] and undergo dietary modification. One of the most widely used rodent dietary NASH-HCC models is a non-genetically modified model of Fischer 344 rats fed a choline-deficient, methionine-lowered, L-amino acid-defined diet (CDAA) for a long period of time [15–17]. This rat CDAA-NASH model has provided useful information to understand its human counterpart, such as the role of oxidative stress and signaling abnormalities in NASH and NASH-associated hepatocarcinogenesis [15–17]; however, numerous points remain unclear. Because mice are largely resistant to CDAA [18], Matsumoto et al. recently developed a modified CDAA that can induce NASH in mice with reduced methionine and a high-fat content (termed as CDAHFD) [19]. We have also developed a different mouse model with a choline-deficient, methionine-lowered, L-amino acid-defined, high-fat diet containing shortening without *trans* fatty acids (CDAA-HF-T[−]); this diet induces fibrosis and proliferative lesions in the liver within 6 months [20].

This study aimed to develop a mouse CDAA-HF-T(−) model capable of assessing NASH-associated hepatocarcinogenesis and identifying key signaling factors involved in its underlying mechanisms.

## Materials and methods

### Diets

The control groups were fed the CE-2 diet, which contained 58% carbohydrate, 13% fat, and 29% protein on a caloric basis, as well as 0.21% choline and 0.44% methionine (CLEA Japan Inc., Tokyo, Japan). The experimental groups were fed the CDAA-HF-T(−) diet (45 kcal% fats from shortening without *trans* fatty acids, Primex Z^®^, and 0.1% methionine), which was a made-to-order product (ID: A16032902) from Research Diet Inc. (New Brunswick, NJ, USA). The components of these diets were described elsewhere [20].

### Animals

Eighty 5-week-old male C57BL/6J mice were purchased from Japan SLC (Shizuoka, Japan) and acclimated for a week before the study. During the acclimation and experimental periods, they were maintained under temperature-controlled conditions (22°C) in colony cages with a 12-hour light/12-hour dark cycle and free access to food and water. At 6 weeks of age, the mice were randomly assigned to four groups of 20 animals each (52- and 78-week control groups, and 52- and 78-week CDAA-HF-T(−) groups). However, because several CDAA-HF T(−)-fed animals died during Week 63, all the remaining mice in the scheduled 78-week sacrifice groups were necropsied at the end of Week 63. Body weight, food consumption, and water intake were all monitored weekly. To confirm the reproducible frequency of tumor incidence, a second examination was performed under the same conditions for the longer examinations. For the second study, control and CDAA-HF-T(−) groups of 20 animals each were fed from 6 to 63 weeks. For **t**he short-term experiment, six animals per group were reared for 13 weeks in the control and CDAA groups, and the samples obtained were used for immunohistochemical staining and quantitative real-time polymerase chain reaction (qPCR). Experimental conditions were maintained according to those previously reported [20]. At the end of the experiment, blood samples were collected from the tail veins of all mice. The mice were then euthanized by exsanguination under light isoflurane anesthesia. During the autopsy, all organs were carefully examined, and the liver and white adipose tissue were excised and weighed. The liver lobes were weighed separately according to the International Harmonization of Nomenclature and Diagnostic (INHAND) criteria [21], with the left lateral lobe, right and left medial lobes, and the rest of the lobes constituting the “others” group. The macroscopic photographs of the liver were then recorded, and the number and size of the masses were measured.

### Histopathological analysis

The liver samples were fixed in 10% neutrally buffered formalin, embedded in paraffin, and cut into 4-μm-thick sections for hematoxylin and eosin (H&E), and Sirius red staining. Using Sirius Red-stained specimens, the areas of fibrosis were measured using a cellSens Dimension software (Olympus, Tokyo, Japan). The liver lesions were histopathologically diagnosed by a scientist blinded to the mouse treatment and according to the following INHAND criteria [21]: regenerative hepatocellular hyperplasia (lesions spanning several hepatic lobules, with portal triads and central veins), hepatocellular adenoma (HCA; lesions greater than several lobules, with no portal triads or central veins), and HCC (lesions containing hepatocytes, with trabecular multiple cell layers or a pseudoglandular appearance). The hepatocyte architecture was confirmed using silver impregnation staining. In addition to the hepatocellular tumor, the following lesions were identified: hemangiosarcoma (lesions forming nodules with an irregular vascular cavity and containing endothelial lining cells with moderate pleomorphism and multilayered clusters) and cholangiofibrosis (lesions containing dilated-to-cystic bile ducts filled with mucus and cellular debris and surrounded by inflammatory cell infiltrates and connective tissue). To improve the quality of the pathology data, our findings and diagnosis were peer-reviewed by a board-certified toxicologic pathologist.

As described elsewhere [22], 13-week and 63-week mouse samples were used for immunohistochemical analyses. The following primary antibodies were used: rat anti-mouse monoclonal antibody for CD68 as a macrophage marker (Abcam, Cambridge, UK), mouse anti-proliferating cell nuclear antigen (PCNA) as a marker of cellular proliferation (DAKO, Kyoto, JPN) and rabbit anti-rat monoclonal antibody for macrophage inflammatory protein 2 (MIP-2; Thermo Fisher Scientific, CA, USA). Heat-mediated antigen retrieval was performed in citrate buffer (pH10mM, 6.0) in a microwave oven (600W 10min) for CD68 and PCNA or an autoclave (121°C 10min) for MIP-2. Antibody binding was visualized using a Histofine Simple Stain Kit (Nichirei Corp., Tokyo, Japan). PCNA-positive cells were counted in 5 randomly selected fields while viewing each slide at ×200 magnification. The average cell number in each group was calculated.

### Biochemical assay

The plasma was separated from blood samples, and alanine (ALT) and aspartate (AST) transaminase activities were measured using an automated chemistry analyzer (DRI-CHEM; Fujifilm, Tokyo, Japan).

### RNA extraction and analysis

Total RNA was extracted from liver tissue samples using Sepasol reagent (Nacalai Tesque, Kyoto, Japan) and reverse transcribed using the PrimeScript RT Master Kit (Takara Bio Inc., Shiga, Japan) according to the manufacturers’ instructions. Thereafter, qPCR was performed using SYBR Premix Ex Taq (Takara Bio Inc., Shiga, Japan) and specific primer sets on a Thermal Cycler Dice Real-Time System Single (Takara Bio Inc., Shiga, Japan). S1 Table lists the primer sequences used for qPCR. The mRNA expression levels were normalized to those of 36B4 mRNA. Then, portions of the RNA samples were subjected to RNA sequencing (RNA-Seq) and corresponding qPCR analyses. RNA-Seq was performed as previously described [23]. For the library preparation, 100 ng of RNA samples from the liver tissue of the control-diet-fed mice (control), as well as nontumoral (CDAA-HF-T[−]-N) and tumoral (CDAA-HF-T[−]-T) liver tissues of the CDAA-HF-T(−)-fed mice (diet treatment for 63 weeks, *n* = 3–6) were obtained. The sequencing libraries were generated using the TruSeq RNA Library Preparation Kit v2 (Illumina Inc., San Diego, CA, USA). Moreover, the principal component analysis, differential expression analysis, heat map generation with hierarchical clustering of samples, and feature and functional annotation analyses were performed using the Ingenuity Pathway Analysis (IPA) software (Ingenuity Systems, Qiagen Co., Ltd.) as previously described [23]. The IPA for the 13-week was reanalyzed the RNA-Seq data used in the previously reported [20].

### Statistical analysis

The numerical values were expressed as means and standard deviations. The statistical differences between groups were evaluated using one-way analysis of variance followed by the Tukey–Kramer test. The incidences of lesion occurrence were statistically tested using Pearson’s chi-square test. All statistical analyses were performed using GraphPad Prism version 8 (GraphPad Software, Inc., CA). Differences were considered significant if the *p*-value was less than 0.05.

### Ethical consideration

All of our animal husbandry and experiments followed the guiding principles of the Tokyo University of Agriculture and were approved by the Animal Experiment Committee of the university (approval no. 2019031). This study complied with all relevant domestic and international laws, regulations, and guidelines. The animal experiments, in particular, complied with the Japanese Act on Welfare and Management of Animals, Standards relating to the Care and Keeping and Reducing Pain of Laboratory Animals, and the ARRIVE (Animal Research: Reporting of *in vivo* experiments) guidelines, as well as the UK Animals (Scientific Procedures) Act 1986 and associated guidelines, EU Directive 2010/63/EU for animal experiments, and the National Institutes of Health guide for the care and use of laboratory animals (NIH Publications No. 8023, revised 1978). Our previous research clearly demonstrated that female animals are resistant to CDAA [24]; hence, only male mice were used in this study.

## Results

### Body weight changes and survival rate

Compared to the control group, the body weight of the CDAA-HF-T(−) group transiently decreased during the first 20 weeks and then recovered into the same range, but it decreased again at the end of Week 45 (S1A Fig).

Animals in the CDAA-HF-T(−) group began to die at the end of Week 40, and the number of dead or moribund animals substantially increased by the end of Week 60 (S1B Fig), forcing us to terminate the experiment at the end of Week 63 by sacrificing all surviving mice. Since multiple nodules were observed in the liver in all cases, severe hepatic change was assumed to be the cause of death. Consequently, 18 and 15 animals from 52- and 63-week CDAA-HF-T(−) groups, respectively, were used in subsequent analyses. In the second study, the mortality status of the CDAA-HF-T(−) group was the same as in the first study, with 15 of 20 animals surviving and used for analysis. Meanwhile, the number of animals in the control group at each time point remained constant at 20.

### Final body and organ weights and plasma biochemistry

At 52 and 63 weeks, the final body weights were significantly lower in the CDAA-HF-T(−) groups than in the control groups (Table 1). However, at both time points, the absolute and relative liver weights were dramatically higher in the CDAA-HF-T(−) groups than in the control groups. In the CDAA-HF-T(−) groups, the liver was heavier at the end of Week 52 than at the end of Week 63 (Table 1). In terms of lobular difference, the absolute and relative weights of the left lateral, medial (only at the end of Week 63), and other lobes were significantly higher in the CDAA-HF-T(−) groups than in the control groups at both time points. In the CDAA-HF-T(−) groups, the lobes were heavier at the end of Week 63 than at the end of Week 52, with the weight gain in the other lobes being the greatest (S2 Table). Accordingly, the CDAA-HF-T(−) groups had significantly higher plasma ALT and AST activities than the control groups at both time points. In the CDAA-HF-T(−) groups, the ALT activity was higher at the end of Week 63 than at the end of Week 52 (Table 1).

**Table 1:** Organ weights and plasma and hepatic chemistries at the ends of Weeks 52 and 63.

Experiment 1.				
	Control 52w	CDAA-HF-T(−) 52w	Control 63w	CDAA-HF-T(−) 63w
BW (g)	34.36 ± 1.63	30.54 ± 4.36*	35.26 ± 1.95	29.63 ± 2.42*
Liver (g)	1.2 ± 0.07	3.0 ± 1.3*	1.27 ± 0.08	4.11 ± 0.79*^,^ **
Liver/BW (%)	3.5 ± 0.12	9.77 ± 4.01*	3.62 ± 0.14	13.65 ± 3.55*^,^ **
eWAT (g)	0.96 ± 0.19	1.0 ± 0.59	0.92 ± 0.3	0.77 ± 0.24* **
eWAT/BW (%)	2.77 ± 0.46	2.81 ± 1.68	2.61 ± 0.77	2.54 ± 0.81
ALT (U/L)	22.2 ± 5.16	299.4 ± 202.69*	36.95 ± 31.11	448.25 ± 398.83*^,^ **
AST (U/L)	44 ± 4.32	358.28 ± 214.91*	60.5 ± 27.62	435 ± 409.53*

Experiment 2		
	Control 63w	CDAA-HF-T(−) 63w
BW (g)	35.67 ± 2.30	30.00 ± 2.78*
Liver (g)	1.39 ± 0.12	4.81 ± 1.22*
Liver/BW (%)	3.89 ± 0.21	15.91 ± 3.12*
eWAT (g)	0.96 ± 0.25	0.59 ± 0.24*
eWAT/BW (%)	2.67 ± 0.56	1.91 ± 0.71*
ALT (U/L)	21.15± 5.03	335.67 ± 247.76*
AST (U/L)	96.95 ± 37.10	511.67 ± 428.21*

*Note*. eWAT, epididymal white adipose tissue. Values are the mean ± *SD*.

*Significantly different from the control value.

**Significantly different from the CDAA-HF-T(−) 52w value.

The absolute weight of white adipose tissue was comparable between the CDAA-HF-T(−) and control groups at the end of Week 52, but it decreased at the end of Week 63 in the CDAA-HF-T(−) groups. Meanwhile, the relative weights did not differ between both groups (Table 1).

### Gross findings

**Fig 1:**
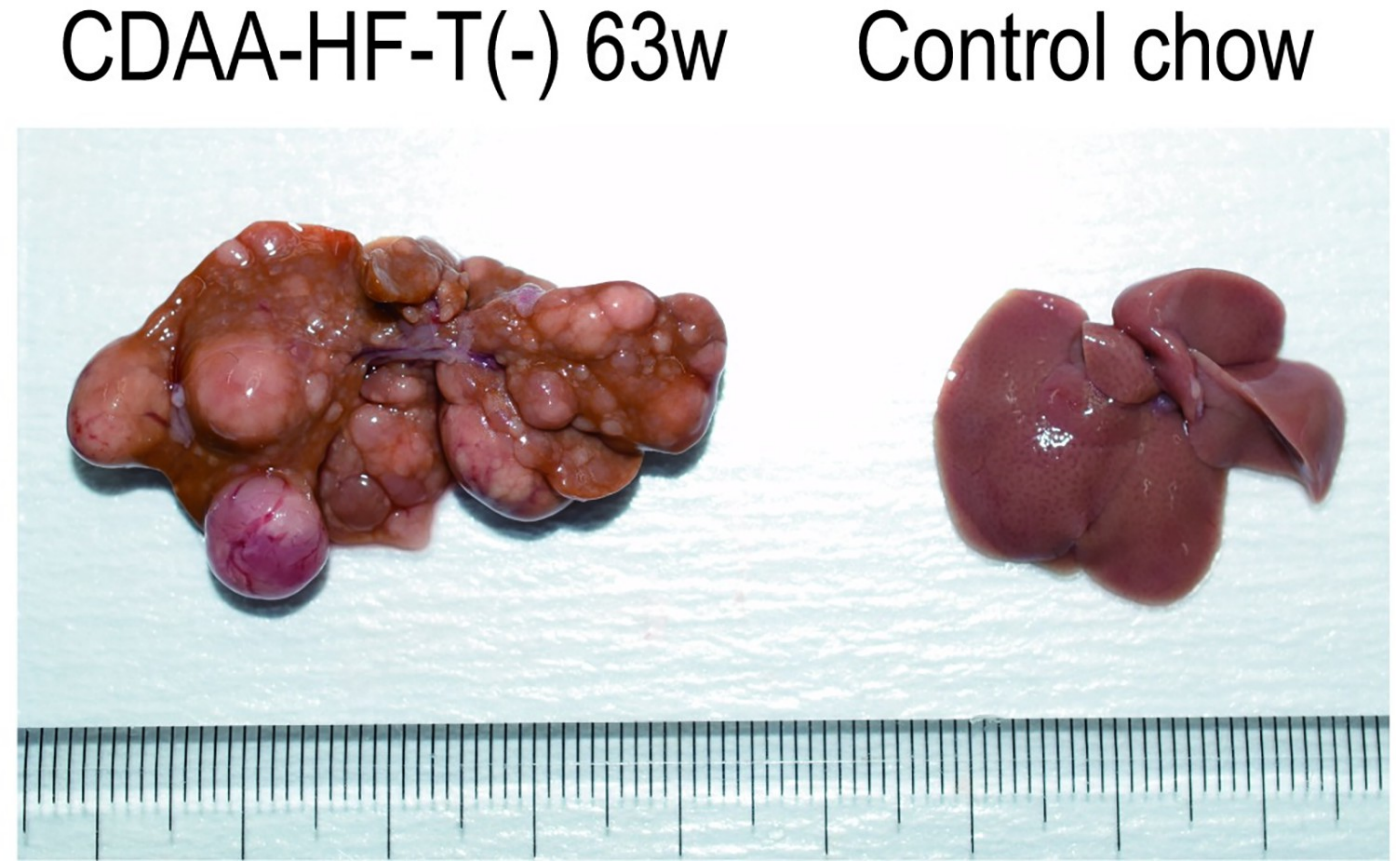
Gross findings in C57BL/6J mice fed with the control chow diet and CDAA-HF-T(−) at the end of Week 63. The representative macroscopic appearances of the liver samples at the end of Week 63. CDAA-HF-T(−), choline-deficient, methionine-depleted, L-amino-acid-defined, high-fat diet containing shortening without *trans* fatty acids.

In both the 52- and 63-week CDAA-HF-T(−) groups, multiple nodules of varying sizes were macroscopically detected in the liver, occasionally in association with hemorrhage (Fig 1), with 0, 1–10, and 7–17 nodules being detected in a mouse from the control, 52-week CDAA-HF-T(−), and 63-week CDAA-HF-T(−) groups, respectively (S2A Fig). In each CDAA-HF-T(−)-fed mouse, the maximum diameter of the nodules was 6–19 mm at Week 52 and 9–19 mm at Week 63 (S2B Fig), with the greatest number of nodules existing in all hepatic lobes except the left lateral and medial ones (S2C Fig).

### Histological findings

**Fig 2:**
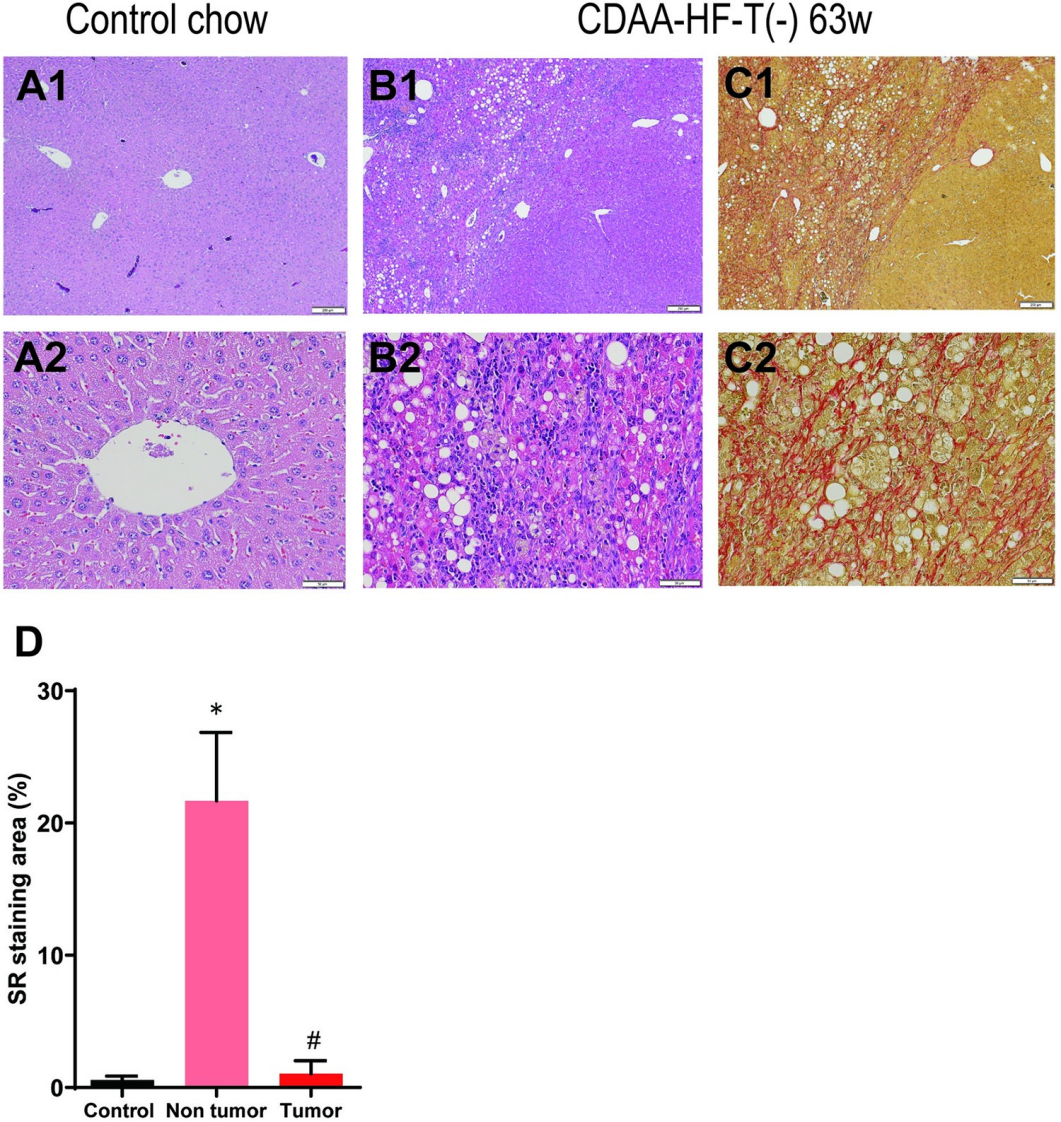
Representative histopathology of the mouse liver at the end of Week 63. Low-power original magnification ×40 (A1, B1, and C1). High-power original magnification ×200 (A2, B2, and C2). Severe lobular inflammation, fibrosis, and numerous foamy macrophages were observed in the liver samples of mice fed with CDAA-HF-T(−) but not of those fed with the control diet. Comparison of Sirius Red-stained areas in control, nontumoral liver area and tumoral liver area of the CDAA-HF-T(−) (D). Results are expressed as percentage of section staining (+) for Sirius Red. *Significantly different from the control group value. #Significantly different from the CDAA-HF-T(−)-Non tumor value.

Fig 2 shows representative histopathology in the nontumoral area of the mouse liver at the end of week 63. Severe lobular inflammation, fibrosis, and numerous foamy macrophages were observed in the liver samples of mice fed with CDAA-HF-T(−), but not of those fed with the control diet. (Fig 2 A1–C2). The magnitudes of Sirius Red-positive area were dramatically elevated in the nontumoral area of the CDAA-HF-T(−)-fed mice liver but not the tumoral area (Fig 2D). Similar changes were already observed at the end of week 52.

**Fig 3:**
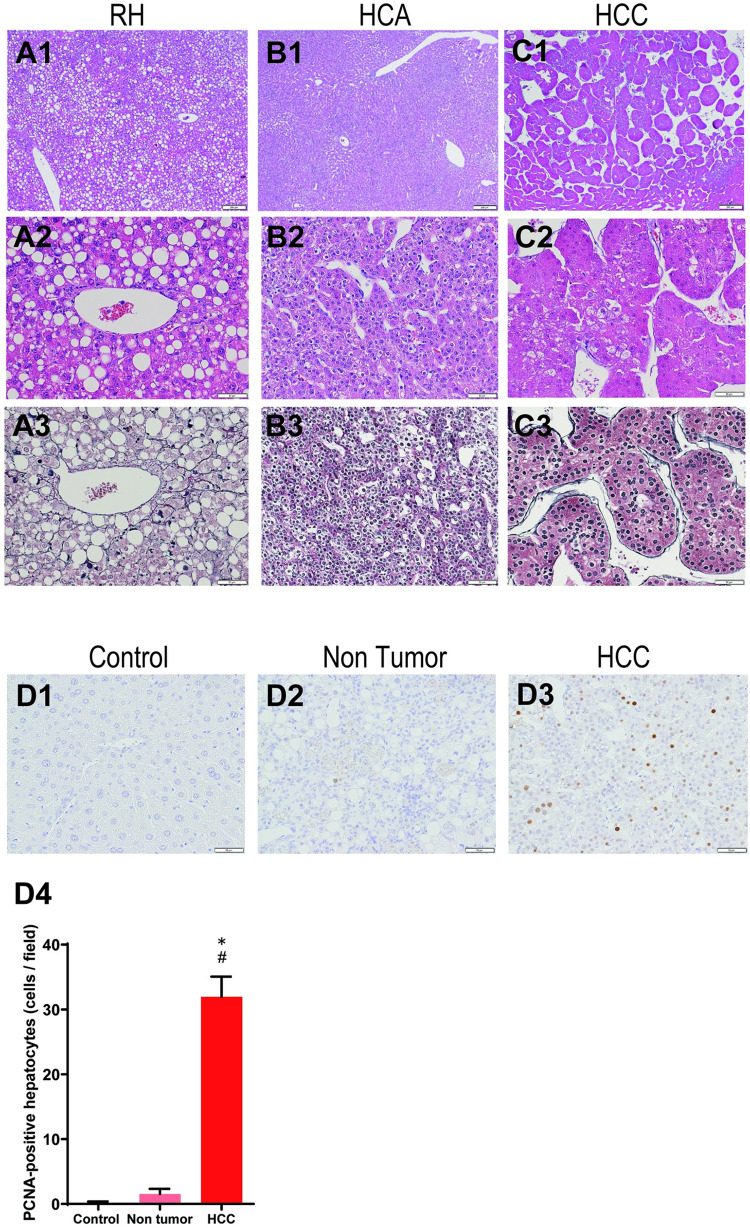
Representative histopathology of the proliferative liver lesions at the end of Week 63. Representative liver for H&E staining that shows RH (A1 and A2), HCA (B1 and B2), and HCC (C1 and C2). Silver impregnation staining confirmed the hepatocyte architectures of RH (A3), HCA (B3), and HCC (C3). The average number of PCNA-positive cells was counted in in control, nontumoral liver area and HCC area of the CDAA-HF-T(−) (D1-4). *Significantly different from the control group value. #Significantly different from the CDAA-HF-T(−)-Non tumor value. Low-power original magnification ×40 (A1, B1, and C1). High-power original magnification ×200 (A/B/C2, A/B/C3 and D). H&E, hematoxylin and eosin staining; HCA, hepatocellular adenoma; HCC, hepatocellular carcinoma; RH, regenerative hyperplasia.

**Fig 4:**
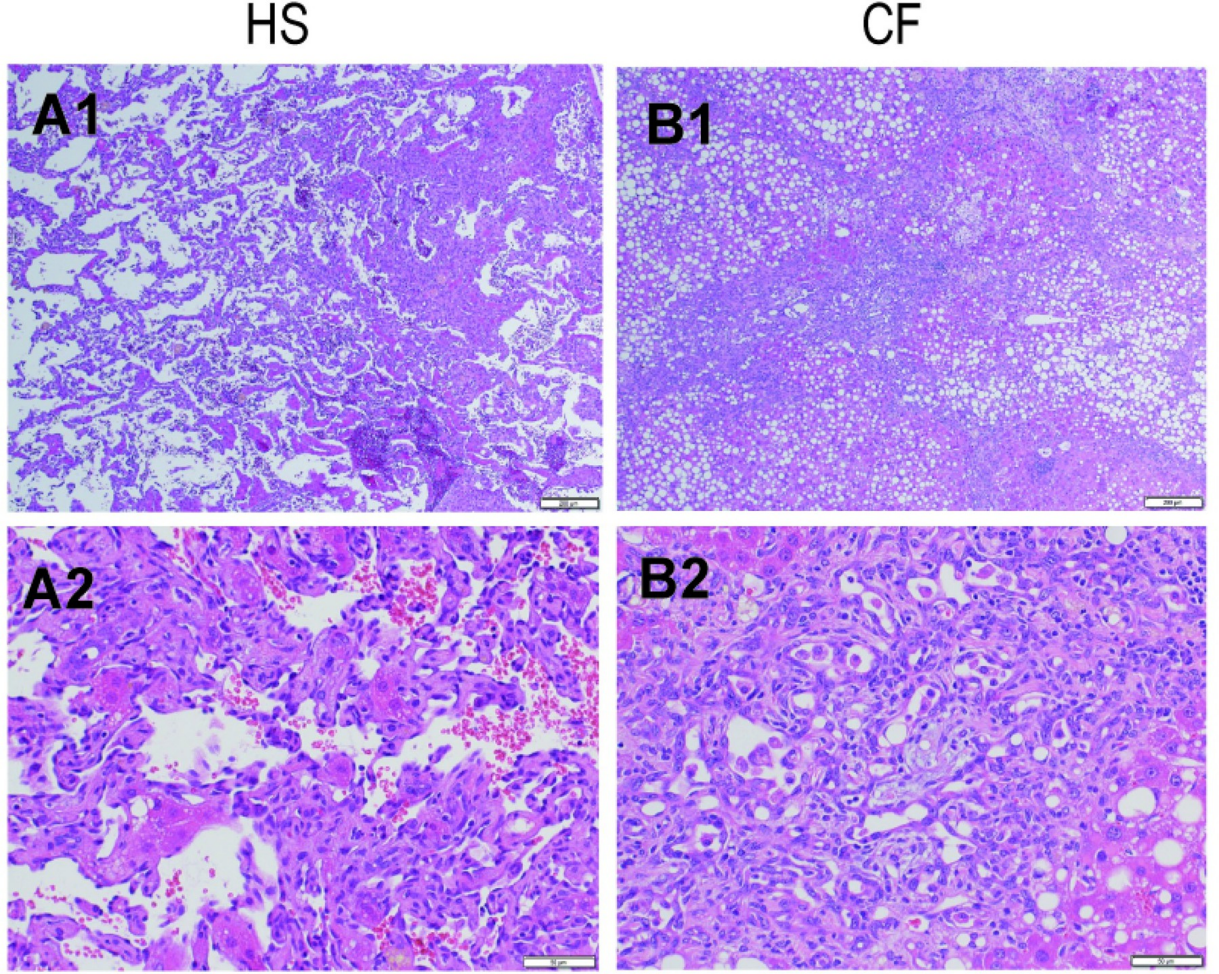
Representative histopathology of the hemangiosarcoma and cholangiofibrosis at the end of Week 63. Representative liver for H&E staining that shows HS (A1 and A2) and CF (B1 and B2). Low-power original magnification ×40 (A1 and B1). High-power original magnification ×200 (A2 and B2). CF, cholangiofibrosis; H&E, hematoxylin and eosin staining; HS, hemangiosarcoma.

Figs 3 and 4 represent the histopathology of hepatic nodular and proliferative lesions in the CDAA-HF-T(−) group, and Table 2 lists the incidences of these lesions in all groups. There were no nodular or proliferative lesions in the control group. All of the CDAA-HF-T(−)-fed mice developed regenerative hepatocellular hyperplasia. The hyperplasia compressed the adjacent parenchyma and/or bulged from the surface, but the lobular architecture in the lesion with central veins and portal triads remained unchanged (Fig 3A). The CDAA-HF-T(−) group also developed HCA, with incidence rates of 67% and 93% at the end of Weeks 52 and 63, respectively. The adenoma compressed the adjacent parenchyma and/or bulged from the surface, the lobular architecture in the lesion was lost, and the hepatocyte cords contained multiple cell layers (Fig 3B). Both the 52- and 63-week CDAA-HF-T(−) groups had HCCs, with incidence rates of 22% and 33% at the end of Weeks 52 and 63, respectively. The carcinoma had severe cellular atypia and complete loss of the lobular architecture, with trabecular and pseudoglandular appearances (Fig 3C). Silver staining confirmed the structure and cell layers of the liver lobular architecture (Fig 3A–3C). PCNA-positive cells showed a slightly elevated in the non-tumor areas of the CDAA-HF-T(-) group compared to controls. Conversely, positive cells were frequently found in HCCs of the CDAA-HF-T(-) group (Fig 3D), suggesting that hepatocellular proliferation is elevated in the HCC area. In the second experiment, the incidence rates were similar to the first experiment; RH, HCA, and HCC were 100%, 100%, and 40%, respectively (Table 2).

**Table 2:** Microscopic findings in the liver of C57BL/6J mice at the end of Weeks 52 and 63.

Experiment 1				
	Control 52w	CDAA-HF-T(−) 52w	Control 63w	CDAA-HF-T(−) 63w
RH	0% 0/20	100% 18/18*	0% 0/20	100% 15/15*
HCA	0% 0/20	67% 12/18*	0% 0/20	93% 14/15*
HCC	0% 0/20	22% 4/18*	0% 0/20	33% 5/15*
HS	0% 0/20	0% 0/18	0% 0/20	26% 4/15*
CF	0% 0/20	6% 1/18	0% 0/20	26% 4/15*

Experiment 2		
	Control 63w	CDAA-HF-T(−) 63w
RH	0% 0/20	100% 15/15*
HCA	0% 0/20	100% 15/15*
HCC	0% 0/20	33% 5/15*
HS	0% 0/20	13% 2/15
CF	0% 0/20	40% 6/15*

*Significantly different from the control value.

In the CDAA-HF-T(−) group, hemangiosarcoma and cholangiofibrosis were also observed, with incidence rates of 26% and 26%, respectively, at the end of Week 63. In the second experiment, the incidence rates of hemangiosarcoma and cholangiofibrosis were 13% and 40%, respectively. Hemangiosarcoma exhibited a variety of vascular patterns with multilayered and/or clustered endothelial cells (Fig 4A). Meanwhile, cholangiofibrosis showed marked pericholangial fibrosis, with biliary epithelial proliferation and metaplastic changes in the glandular epithelium (Fig 4B).

**Fig 5:**
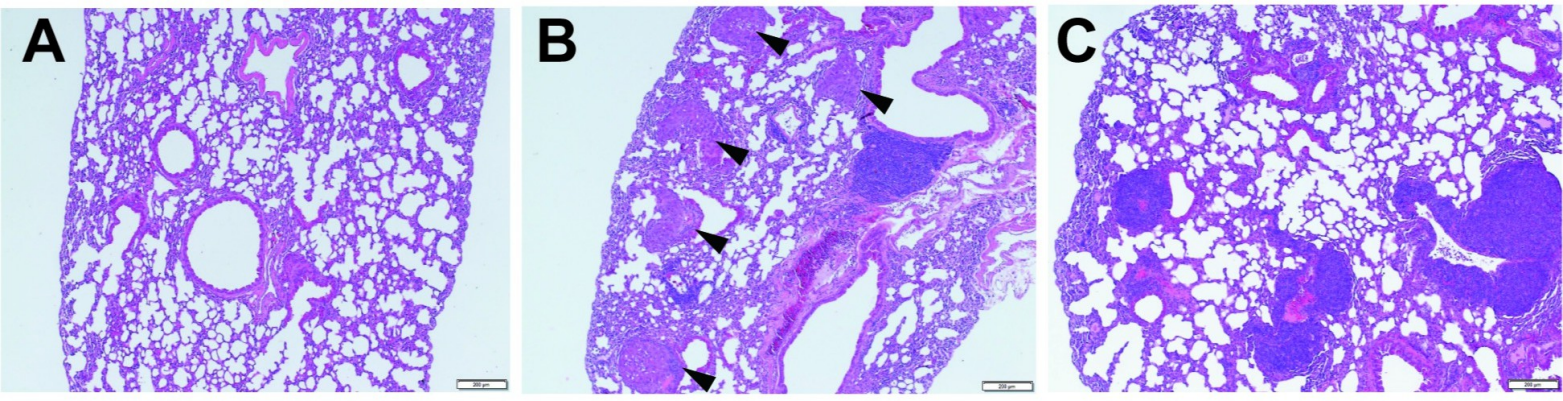
Representative histopathology of the mouse lung at the end of Week 63. Representative liver for H&E staining that shows control (A) and CDAA-HF-T(−) (B and C). (B) Multiple metastatic nodules. (C) Lymphoid clusters. Original magnification ×40.

Metastases in the lungs were examined. The control group yielded no specific results (Fig 5A). In the first experiment, lung metastasis was not observed in all mice. In the second experiment, multiple metastatic nodules were noted in the lungs of one of the animals (Fig 5B). In the lungs of mice in which metastases were found, the number of metastases was fourteen as far as could be observed by the examination of histology. Another finding was the presence of lymphoid clusters in the bronchi/bronchioles of the lung in all CDAA-HF-T(−) group mice both the first and second experiment (Fig 5C).

### Gene expression profiles

**Fig 6:**
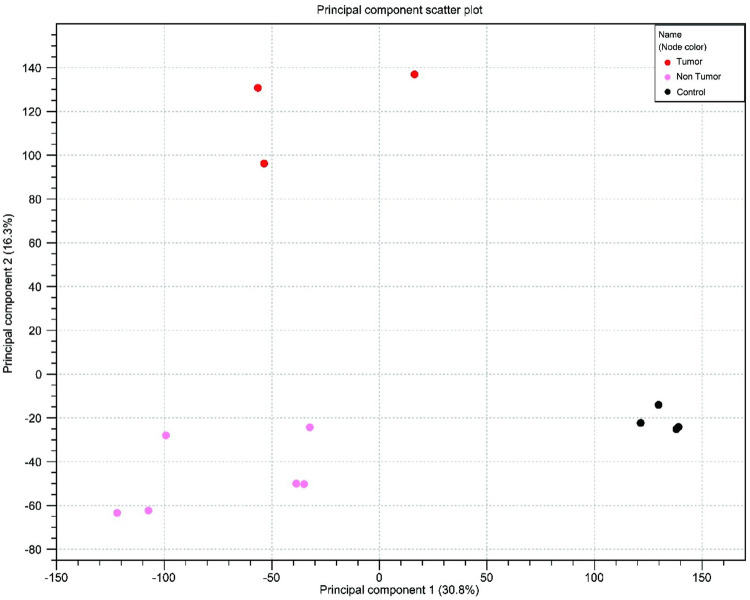
Two-dimensional principal component plot of principal component analysis for RNA-seq. The X axis indicates the first principal component, whereas the Y axis depicts the second principal component. The value after the principal component identifier refers to the proportion of variance explained by this particular principal component.

At the end of Week 63, RNA-Seq was performed using RNA samples obtained from the liver tissue of control-diet-fed mice (control) as well as nontumoral (CDAA-HF-T(−)-N) and tumoral (CDAA-HF-T(−)-T) liver tissues of the CDAA-HF-T(−)-fed animals. Using principal component analysis, outlier samples were identified for quality control, and the primary causes of dataset variation were determined (Fig 6). The first component (30.8%, horizontal axis) separated the control and CDAA-HF-T(−) groups, while the second component (16.3%, vertical axis) separated the CDAA-HF-T(−)-N and CDAA-HF-T(−)-T groups. Several differentially expressed genes (DEGs) were identified in the control, CDAA-HF-T(−)-N, and CDAA-HF-T(−)-T groups with a false discovery rate of p-values less than 0.05 and a fold change greater than 2. The control versus CDAA-HF-T(−)-N, the control versus CDAA-HF-T(−)-T, and the CDAA-HF-T(−)-N versus CDAA-HF-T(−)-T had 5654, 5609, and 2896 DEGs, respectively.

The altered pathways in the development of NASH-related non-neoplastic and neoplastic lesions were specifically identified by conducting a functional analysis of the DEGs using IPA between the control, CDAA-HF-T(−)-N, and CDAA-HF-T(−)-T groups. Tables 3–5 summarize the top 10 upregulated and downregulated pathways with a z score greater than 2 in the IPA of the canonical pathway. IPA according to -log (P-value) based on DEGs. In CDAA-HF-T(−)-N versus control, the upregulated signaling pathways included those related to liver fibrosis, RhoGTPases, which are involved in cell proliferation and survival [25,26], platelet glycoprotein VI, Tec kinase, and estrogen receptor signaling (Table 3). In contrast, the downregulated signaling pathways included those related to RhoGTPases inhibitors, attenuation of amino acid degradation (e.g., tryptophan and isoleucine) and lipid metabolism (e.g., fatty acid β-oxidation and synthesis), and peroxisome proliferator-activated receptors (PPARs) (Table 3). In CDAA-HF-T(−)-T versus control, the upregulated signaling pathways included those related to hepatic fibrosis, inflammatory cytokines (mainly interleukin (IL)-8), chemokines, and exogenous chemical metabolism involving the aryl hydrocarbon receptor (Table 4). In contrast, the downregulated signaling pathways included those related to lipid metabolism-associated signaling pathways, such as the phosphatase and tensin homolog deleted from chromosome 10 (PTEN), a well-known tumor suppressor (Table 4). In CDAA-HF-T(−)-T versus CDAA-HF-T(−)-N, the upregulated signaling pathways included those related to exogenous chemometabolism, such as the constitutive androstane receptor and aryl hydrocarbon receptor, IL-8, hypoxia-inducible factor-1α, and estrogen biosynthesis. In contrast, the downregulated signaling pathways included those related to tumor suppressors such as PTEN and transducers of human epidermal growth factor receptor 2 (TOB) (Table 5).

**Table 3:** Upregulated and downregulated genes in the canonical pathway, CDAA-HF-T(−)-N versus control.

Upregulated	-log(*p*-value)	*z* score
Cardiac hypertrophy signaling (enhanced)	11	8.12
Colorectal cancer metastasis signaling	7.39	6.671
Hepatic fibrosis signaling pathway	6.73	6.602
Role of NFAT in cardiac hypertrophy	4.59	6.49
Synaptogenesis signaling pathway	4.78	6.405
Estrogen receptor signaling	3.71	6.405
Signaling by rho family GTPases	3.57	6.337
Systemic lupus erythematosus in B cell signaling pathway	7.95	5.97
Tec kinase signaling	4.2	5.84
GP6 signaling pathway	6.62	5.814
Downregulated	-log(*p*-value)	*z* score
LXR/RXR activation	2.99	−4.95
Rho GDI signaling	3.13	−3.904
Tryptophan degradation III (eukaryotic)	4.43	−3.317
Antioxidant action of vitamin C	2.62	−3.157
Fatty acid β-oxidation I	5.14	−3.153
PPAR signaling	3.33	−2.959
Glutaryl-CoA degradation	3.19	−2.646
Endocannabinoid cancer inhibition pathway	2.43	−2.596
Triacylglycerol degradation	1.83	−2.324
Isoleucine degradation I	1.82	−2.236

**Table 4:** Upregulated and downregulated genes in the canonical pathway, CDAA-HF-T(−)-T versus control.

Upregulated	-log(*p*-value)	*z* score
Neuroinflammation signaling pathway	7.01	6.328
IL-8 signaling	8.38	6.078
Hepatic fibrosis signaling pathway	10.3	6.061
Xenobiotic metabolism AHR signaling pathway	7.24	5.425
Xenobiotic metabolism general signaling pathway	5.5	5.397
Role of hypercytokinemia/hyperchemokinemia in the pathogenesis of influenza	3.02	5.292
HIF1α signaling	6.04	5.17
Production of nitric oxide and reactive oxygen species in macrophages	7.52	5.08
Fcγ receptor-mediated phagocytosis in macrophages and monocytes	9.12	5.032
Systemic lupus erythematosus in B cell signaling pathway	6.08	4.939
Downregulated	-log(*p*-value)	*z* score
Rho GDI signaling	6.38	−3.501
LPS/IL-1-mediated inhibition of RXR function	20.3	−3.244
PPAR signaling	4.22	−2.874
SPINK1 pancreatic cancer pathway	0.932	−2.84
PTEN signaling	4.31	−2.667
Antioxidant action of vitamin C	4.22	−2.121

**Table 5:** Upregulated and downregulated genes in the canonical pathway, CDAA-HF-T(−)-T versus CDAA-HF-T(−)-N.

Upregulated	-log(*p*-value)	*z* score
Nicotine degradation II	5.64	3.9
Xenobiotic metabolism CAR signaling pathway	4.23	3.773
Superpathway of melatonin degradation	5.02	3.771
IL-8 signaling	0.611	3.578
HIF1α signaling	1.38	3.53
Estrogen biosynthesis	6.59	3.5
Melatonin degradation I	4.3	3.5
Acetone degradation I (to methylglyoxal)	5.17	3.464
Xenobiotic metabolism AHR signaling pathway	1.14	3.464
Xenobiotic metabolism PXR signaling pathway	3.07	3.413
Downregulated	-log(*p*-value)	*z* score
PTEN Signaling	0.488	−2.828
LPS/IL-1 mediated inhibition of RXR function	8.98	−2.357
Antiproliferative role of TOB in T cell signaling	0.578	−2.236
PPAR signaling	0	−2.121
Dopamine receptor signaling	0.611	−2
Cell cycle: G1/S checkpoint regulation	0	−2

S3–S5 Tables summarize the top 10 upregulated and downregulated pathways with a z score greater than 2 in the IPA of the upstream regulator. In CDAA-HF-T(−)-N and CDAA-HF-T(−)-T versus control, the upstream regulators associated with inflammation and fibrosis such as tumor necrosis factor, Interleukin-1β, Interferon gamma and Transforming growth factor β1 were upregulated (S3 and S4 Tables). Furthermore, CDAA-HF-T(−)-T enhanced the upstream regulators β-estradiol (versus CDAA-HF-T(−)-N or control) and chorionic gonadotropin (S4 and S5 Tables).

The IPA of canonical pathways revealed that the 13-week and 63-week CDAA-HF-T(-) groups had increased RhoGTPases and IL-8 signaling and decreased Liver X receptor (LXR) activity and PPAR signaling compared to controls (S6 Table). In the upstream analyses, the pathways involved in inflammation and fibrosis were also listed (S7 Table).

### Quantitative real-time polymerase chain reaction and immunohistochemical assessments

**Fig 7:**
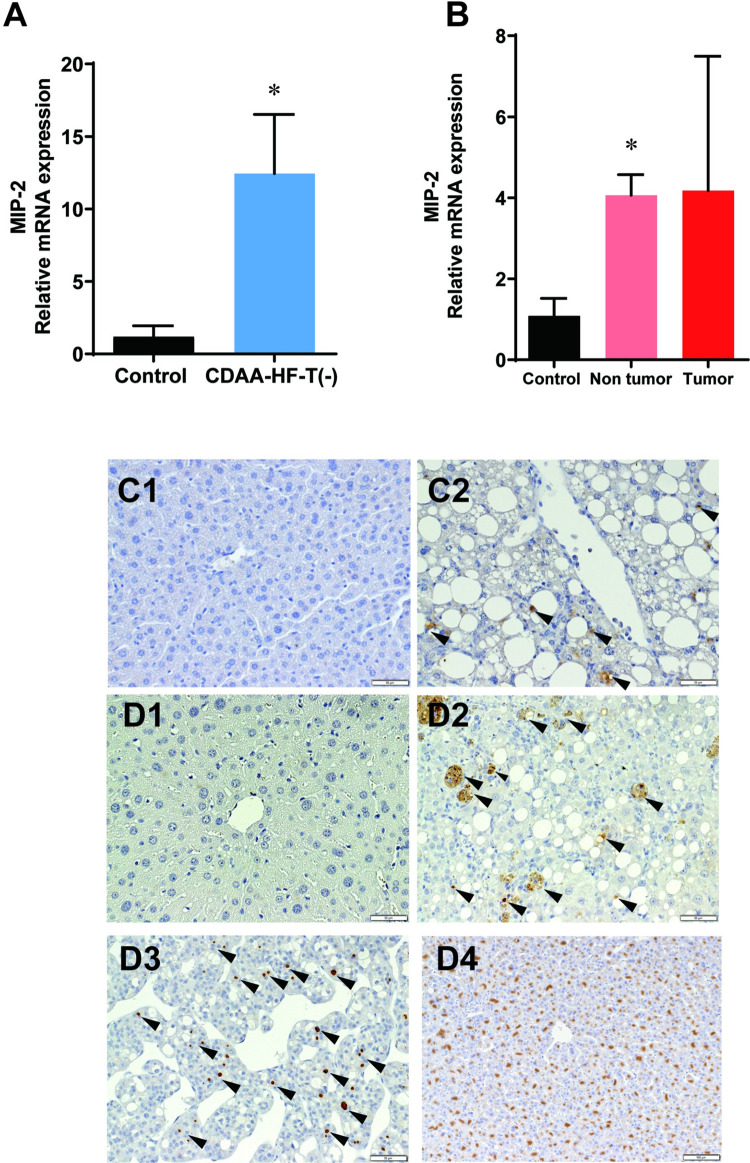
qPCR and immunohistochemical assessments for MIP-2. Quantitative real-time PCR data for genes involved in MIP-2 in the liver samples of mice fed with the control chow or CDAA-HF-T(−) for (A) 13 weeks and (B) 63 weeks. *Significantly different from the control group value. Representative immunohistochemical features for MIP-2 in the liver samples of mice fed with the control chow or CDAA-HF-T(−) for (C) 13 weeks and (D) 63 weeks. Original magnification ×200 (C1,2 D1-3), ×100 (D4). Representative liver for immunostaining that shows control chow (C/D1), CDAA-HF-T(−) nontumoral liver tissues (C/D2), CDAA-HF-T(−) hepatocellular carcinoma (D3), and CDAA-HF-T(−) hepatocellular adenoma (D4). Arrows indicate positive staining. CDAA-HF-T(−), choline-deficient, methionine-depleted, L-amino-acid-defined, high-fat diet containing shortening without *trans* fatty acids.

According to the IPA, IL-8 signaling was upregulated in nontumoral and especially tumoral liver tissues of CDAA-HF-T(−)-fed mice, and the signaling pathway may be involved in NASH and NASH-associated hepatocarcinogenesis. These findings are consistent with a previous study, which found that the IL-8 signaling pathway activates the RhoGTPases and the phosphatidylinositol 3-kinase/protein kinase B signaling pathways [27]. Thus, IL-8 could be a key signaling factor in the background mechanism of the dietary mouse model of NASH. In mice, MIP-2 is thought to be a functional homolog of IL-8 [28,29]. Therefore, we analyzed the changes in MIP-2 gene and protein expressions over time. Meanwhile, *MIP-2* expression levels were significantly higher in the CDAA-HF-T(−) group for 13 weeks than in the control group (Fig 7A). Immunohistochemically, MIP-2 was positive in single small and foamy cells in the liver samples of the CDAA-HF-T(−) group for 13 weeks; however, only a few positive cells were observed in the liver of the control group (Fig 7C1,2). The foamy cells frequently found in the liver samples of CDAA-HF-T(–)-fed mice were CD68 positive (S3 Fig), suggesting their macrophage nature.

In the 63-week CDAA-HF-T(−) group, *MIP-2* expression levels were found to be upregulated in both nontumoral and tumoral liver tissues (Fig 7B). Immunohistochemically, MIP-2 was positive in single small cells and foamy macrophages in nontumoral liver tissues at the end of Week 63, whereas in tumoral tissues, MIP-2 positivity was observed in a portion of the cytoplasm of HCC and HCA (Fig 7D1–7D4).

RT-qPCR was used to verify the CDAA-HF-T(–)-induced reduction in LXR activity and PPAR signaling (S4 Fig). In a 13-week study, RT-qPCR was used to assess the expression of *LXRα* and its target *sterol regulatory element-binding protein-1c* (*SREBP-1c*), *PPARα* and its target *acyl-CoA oxidase* (*ACO*), and *PPARγ2* and its target *fat-specific protein 27* (*FSP27*). *LXR*, *SREBP-1c*, *PPARα*, and *ACO* expression levels were lower in the CDAA-HF-T(−) group than in the control group, indicating decreased fatty acid synthesis and lipid oxidation due to decreased LXRα activity and PPARα signaling, while *PPARγ2* and *FSP27* expression levels were higher. In the 63-week study group, *PPARα* and *ACO* expression levels were lower in the CDAA-HF-T(−) group than in the 13-week study group. Interestingly, *LXRα* expression levels remained unchanged in the control group. However, *SREBP-1c* expression levels were higher in both nontumorous and tumorous tissues in the 63-week CDAA-HF-T(−) group than in the 13-week CDAA-HF-T(−) group, while the *PPARγ2* and *FSP27* expression levels were lower. The nontumorous and tumorous tissues in the CDAA-HF-T(−) group at Week 63 showed comparable results.

## Discussion

This study developed a mouse model of NASH-associated hepatocarcinogenesis using CDAA-HF-T(−), with hepatic tumors such as HCC, HCA, and hemangiosarcoma all induced at a high rate. Metastasis to the lungs was also confirmed in one patient. RNA-Seq and IPA analyses revealed common and distinct profiles of altered signaling factors in the pathogenesis of hepatocarcinogenic and noncarcinogenic NASH lesions. In the CDAA-HF-T(−) group, RhoGTPases and IL-8 signaling were increased in both the tumoral and nontumoral liver tissues, whereas lipid-metabolism-related signaling was decreased. Compared to nontumoral tissues, tumoral liver tissue showed further increases in IL-8 signaling and β-estradiol signaling and a decrease in carcinogenic inhibitor-related signaling. From a relatively early stage of CDAA-HF-T(−) feeding, MIP-2 was upregulated. On the other hand, MIP-2 has expressed on macrophages in non-tumor areas as well as hepatocytes in tumor areas. Thus, IL-8 may contribute to NASH development, but it may also play different roles in inflammation and tumorigenesis.

Long-term CDAA-HF-T(−) feeding induced severe inflammation and fibrosis in nontumoral liver tissue, mimicking the fibrotic and cirrhotic outcomes of the human NASH situation. Nontumoral liver morphology is a common characteristic of CDAA-derived diet-fed rodents, and given its similarity to its human counterpart, the corresponding models could be used for phenomenological and mechanistic studies to assess NASH, NASH-associated hepatocarcinogenesis, and their underlying mechanisms. Although CDAA-fed mice exhibit histological hallmarks of NASH and hypercholesterolemia, the model lacks essential clinical and metabolic features in normal-weight/BMI NASH patients, often characterized using elevated fasting blood glucose, higher rate of diabetes, and hypertriglyceridemia. In contrast to NAFLD, the onset of NASH in humans may not necessarily be correlated to *de novo* lipogenesis, and there is an inhibition of very-low-density lipoprotein synthesis and release from the liver [30], which is similar to the onset of NASH in CDAA-fed rodents [31,32]. This suggest the ensuring models’ usefulness the CDAA-related rodent models and human NASH share many similarities. Therefore, we believe that the currently revealed signal changes are helpful in elucidating mechanisms underlying human NASH with inflammation, fibrosis, and neoplastic lesions.

In our study, the nontumoral liver tissues contained numerous foamy cells surrounded by fibrosis. The foamy cells were CD68 positive, indicating their macrophage nature, but they differed morphologically from resident Kupffer cells or macrophages frequently seen in a crown-like structure in NASH [33]. Although the precise characteristics of foamy macrophages and their roles in NASH-associated fibrosis and cirrhosis are unclear, they may be essential for NASH progression.

In the present study, CDAA-HF-T(−) induced HCC at a high rate. Although dietary NASH-HCC mouse models are seldom reported, high-fat chow containing *trans* fats and high-fructose corn syrup that promotes sedentary behavior in the American-lifestyle-induced obesity syndrome mouse model caused hepatocellular neoplasms after 12 months [34]. Compared to the latter model, the CDAA-HF-T(−) model exhibited multiple macroscopic nodular lesions with severe fibrosis. In our previous report, proliferative lesions were induced in the livers of mice fed CDAA-HF-T(–) for up to 26 weeks, indicating that a proapoptotic hepatic microenvironment is important in the early stages of NASH [20]. Ikawa-Yoshida et al. reported that long-term feeding of CDAHFD, another high-fat CDAA, also induces hepatic tumors with severe fibrosis in the mouse liver [35]. The incidence of macroscopic nodular lesions in our study was higher than in Ikawa-Yoshida et al.’s study, but with similar incidence ranges for HCA and HCC. CDAA-HF-T(–) and CDAHFD are both CDAA-derived high-fat diets with the same methionine content; however, CDAHFD contains 60% kcal of lard, whereas CDAA-HF-T(–) contains 45 kcal of *trans* fatty acid-free shortening. The methionine content may contribute to neoplastic lesion induction, and *trans* fatty acid-free shortening, which mainly contains palmitate acid, may accelerate the formation of macroscopic nodular lesions and cell proliferation. In our study, the incidence and size of macroscopic nodular lesions varied among liver lobes. The distinct lobe hypertrophy is seen in human NASH or alcoholic cirrhosis and could be attributed to blood flow alterations [36].

In this study, the CDAA-HF-T(–) group developed hemangiosarcoma and cholangiofibrosis at the end of Week 63 at the same incidence rate of 26%. These liver proliferative lesions were not mentioned in Ikawa-Yoshida et al.’s report on NASH-associated hepatocarcinogenesis in CDAHFD-fed mice [35], possibly because these researchers were only comparing different diets. Thus, further studies are required. Hemangiosarcoma occurs spontaneously in 2–5% of mice and less than 0.001% of humans, indicating a species difference [37], implying that the hemangiosarcoma in the present study did not develop spontaneously; however, it must be related to CDAA-HF-T(−) feeding. Although hemangiosarcoma is uncommon in humans, it is clinically very malignant and poorly understood. In the human liver, primary hemangiosarcoma is the most common mesenchymal tumor, accounting for approximately 2% of all primary liver tumors [38]. In a Chicago autopsy series, every 30 cases of HCC had primary hepatic angiosarcoma [39]. Our study elucidated the pathogenesis of hepatic primary hemangiosarcoma as well as the relationship between NASH and hemangiosarcoma, contributing to angiosarcoma treatment in humans. Although the mechanism of cholangiofibrosis in hepatocarcinogenesis is unknown, NAFLD/NASH is one of the risk factors for cholangiocarcinoma, which has a poor prognosis in humans [40]. The hepatic NASH signaling microenvironment in CDAA-HF-T(−)-fed mice may induce tumor growth on both hepatocyte and cholangiocyte lineages, resulting in HCA, HCC, and cholangiofibrosis.

In the present study, a case of lung metastasis was observed. Metastasis is extremely uncommon in the diet-induced NASH-HCC model. In mice, we found that CDAA-HF-T(−) dietary administration can cause lung metastasis and HCC. In addition, we found lymphoid clusters in the bronchus/bronchioles of lungs. Under chronic inflammatory conditions, lymphocyte-specific microdomains can be formed as tertiary lymphoid structures in nonlymphoid organs [41]. Tertiary lymphoid structures are sometimes believed to be inducible bronchus-associated lymphoid tissue (iBALT). iBALT is formed in a wide range of diseases, including hepatitis C [42]. Thus, the presence of a cluster of lymphocytes in the lungs suggested that iBALT was caused by chronic liver inflammation. However, further close examination is needed, including the possibility of other diseases, such as lymphoma.

The IPA findings revealed that RhoGTPase signaling was upregulated and lipid-metabolism-related signaling was downregulated in both tumoral and nontumoral liver tissues of CDAA-HF-T(−)-fed mice, with similar findings for the 13-week IPA, implying a role for RhoGTPase signaling in inducing both tumoral and nontumoral NASH lesions. RhoGTPases are involved in a variety of cellular processes, such as cell proliferation, differentiation, motility, adhesion, survival, and secretion [26]. In fact, cell proliferation increased after 13 weeks of CDAA-HF-T(-) feeding, indicating the involvement of RhoGTPases [20]. Although the relationship between RhoGTPases and NASH remains poorly understood, gene profiling of human NASH samples has revealed that RhoGTPase signaling is associated with the progression of liver fibrosis [43]. LXL/RXR and PPARs participate in cellular lipid metabolism as nuclear receptors, and their activation attenuates NASH pathogenesis, implying a protective mechanism against NASH, such as the attenuation of adipose-derived Toll-like receptor 4 activation [44]. In this experiment, the RT-qPCR findings revealed a decrease in nuclear receptor expression. *PPARα* and *ACO* expression levels decreased from Week 13 to Week 63 of CDAA-HF-T(-) feeding, indicating a sustained decrease in PPARα activity. However, *PPARγ2* and *FSP27* expression levels increased at Week 13 and then decreased at Week 63. Importantly, PPARγ2 is expressed in the liver, specifically in hepatocytes, and is critical for the development of NAFLD [45]. Therefore, increased *PPARγ2* and *FSP27* expression levels at Week 13 may indicate an increase in oil droplet synthesis in the liver. However, *PPARγ2* and *FSP27* expression levels decreased at Week 63, implying a decrease in oil droplet synthesis, particularly as nontumorous and tumorous liver tissues developed fibrosis and tumorigenesis. In addition, *LXRα* expression levels decreased at Weeks 13 and 63 in the nontumorous liver tissue, while the expression of SREBP-1c, an LXRα target, decreased at Week 13 but increased at Week 63. Furthermore, *SREBP-1* expression levels were found to be significantly higher in HCC tumors than in adjacent tissues, suggesting that it may be involved in tumorigenesis. *SREBP-1* downregulation inhibited HCC HepG2 and MHCC97L cell proliferation and induced apoptosis [46]. The role of fatty acid synthesis in NASH-related hepatocellular tumorigenesis has yet to be investigated. Therefore, the almost complete downregulation of a series of nuclear receptors observed in this study indicated that such a protective mechanism against NASH failed and played a crucial role in NASH pathogenesis in CDAA-HF-T(−)-fed mice. This phenomenon could be explained by the excess lipids and their subsequent cellular and microenvironmental stresses, which could be extrapolated to the human situation.

The NASH pathology includes hepatic adipogenesis, inflammation, fibrosis, and HCC [47]. TOB is a member of an antiproliferative gene family and acts as a transcriptional corepressor by suppressing cyclin D1 promoter activity *via* histone deacetylase interaction [48,49]. TOB-deficient mice are prone to the spontaneous formation of tumors, such as HCA, hemangiosarcoma, and malignant lymphoma [50]. Estradiol signaling is upregulated in tumoral liver tissue; thus, we previously demonstrated that CDAA-HF-T(−) induced proliferative lesions in the mouse liver at an early stage and that sulfotransferase family 1E member 1, an enzyme that causes estradiol sulfation and inactivation, was markedly upregulated. Estrogen promotes hepatocyte proliferation *via* the G-protein-coupled estrogen receptor [51]. Taken together, the altered signaling changes seen in the tumoral liver tissues of CDAA-HF-T(−)-fed mice may serve as targets for assessing the underlying mechanisms of NASH and NASH-associated hepatocarcinogenesis and seeking mechanism-based disease control.

The IL-8 signaling pathway activates the RhoGTPase and phosphatidylinositol 3-kinase/protein kinase B signaling pathways [24]. This finding is consistent with the present data; hence, IL-8 might be a key signaling factor for NASH and NASH-associated hepatocarcinogenesis. IL-8 is produced by macrophages, epithelial cells, airway smooth muscle cells, and endothelial cells [52] and participates in the induction and amplification of inflammatory processes [53]. *MIP-2* expression was upregulated in the liver of mice fed CDAA-HF-T(−) for 13 weeks, as well as in the nontumoral liver tissue of mice fed such a diet for 63 weeks. It was mostly found in foamy macrophages and single small cells, implying that IL-8 originates from nonparenchymal cells. Because these foamy macrophages were surrounded by fibrosis, IL-8 signaling and foamy macrophages may play an important role in NASH-related liver fibrosis. In humans, serum IL-8 levels correlated with NAFLD prevalence better than other proinflammatory cytokines [54]. Therefore, IL-8 could be a diagnostic and therapeutic target for NASH.

In addition, *MIP-2* expression was upregulated in the tumoral liver tissue of mice fed CDAA-HF-T(−) for 63 weeks. In this study, MIP-2-positive cells were detected in the cytoplasm of some hepatocellular carcinomas and adenomas. Consistent with the present results, previous reports have concluded that IL-8 regulates AKT activation via RhoGTPase activation and PTEN suppression, thus affecting cell survival, proliferation, migration, and angiogenesis in the tumor microenvironment [55]. Furthermore, IL-8 secretion positively correlates with the enrichment of liver cancer stem cells [56], and IL‑8 promotes cell migration in liver cancer [57]. Therefore, IL-8 signaling may be a critical phenomenon of neoplastic cell transformation and should be thoroughly investigated in order to understand and control NASH-associated hepatocarcinogenesis.

## Conclusions

In conclusion, the CDAA-HF-T(−) mouse model is useful for investigating NASH and NASH-associated hepatocarcinogenesis. The model aided in elucidating the underlying mechanisms of such diseases, allowing for the development of therapeutic and preventive measures as well as the investigation of their applicability to human NASH. In this study, we found that the pathogenesis of NASH and NASH-related liver carcinogenesis is continuously activated by several common signals. On the contrary, we also found specific alterations in tumor tissues. We believe that IL-8 signaling is important in all stages of NASH, from initial inflammation to fibrosis progression and tumorigenesis.

## Supporting information

S1 Fig Body weight changes and survival rate. (A) Body weight changes and (B) survival rate of C57BL/6J mice fed with the control chow diet, CDAA-HF-T(−) 52 or 63 weeks.
(TIF)

S2 Fig The interlobar difference of the nodule numbers. The number of the nodules in the left lateral lobes, medial lobes, and others in the liver of mice fed with CDAA-HF-T(−) for 52 and 63 weeks.
(TIF)

S3 Fig Immunohistochemical assessments for the CD68. Representative immunohistochemical features for CD68 in the livers of mice fed with the control chow (A) and CDAA-HF-T(−) (B) for 13 weeks. Arrows indicate positive staining.
(TIF)

S4 Fig qPCR assessments of LXR and PPAR signaling related. Quantitative real-time PCR of genes involved in *LXRα* (A), *SREBP-1c* (B), *PPARα* (C), and *AOX* (D) in the livers of mice fed with the control chow, CDAA-HF-T(−) for 13 weeks (A/B/C/D1) or 63 weeks (A/B/C/D2). *Significantly different from the control group value. Quantitative real-time PCR of genes involved in *PPARγ2* (E) and *FSP27* (F) in the livers of mice fed with the control chow, CDAA-HF-T(−) for 13 weeks (E/F1) or 63 weeks (E/F2). *Significantly different from the control group value.
(TIF)

S1 Table Sequence information of primers for the quantitative real-time PCR analysis. (DOCX)

S2 Table Liver lobe weights at the ends of Weeks 52 and 63. (DOCX)

S3 Table Upregulated and downregulated genes in the upstream regulator, CDAA-HF-T(−)-*N* versus control. (DOCX)

S4 Table Upregulated and downregulated genes in the upstream regulator, CDAA-HF-T(−)-T versus control. (DOCX)

S5 Table Upregulated and downregulated genes in the upstream regulator, CDAA-HF-T(−)-T versus CDAA-HF-T(−)-*N*. (DOCX)

S6 Table Upregulated and downregulated genes in the canonical pathway, CDAA-HF-T(−) versus control, 13 weeks. (DOCX)

S7 Table Upregulated and downregulated genes in the upstream regulator, CDAA-HF-T(−) versus control, 13 weeks. (DOCX)
