# Supplementary figures and images for: Nonalcoholic steatohepatitis-associated hepatocarcinogenesis in mice fed a modified choline-deficient, methionine-lowered, L-amino acid-defined diet and the role of signal changes
Source: PLoS One. 2023 Aug 3;18(8):e0287657. doi: 10.1371/journal.pone.0287657 (PMC10399772; doi:10.1371/journal.pone.0287657)

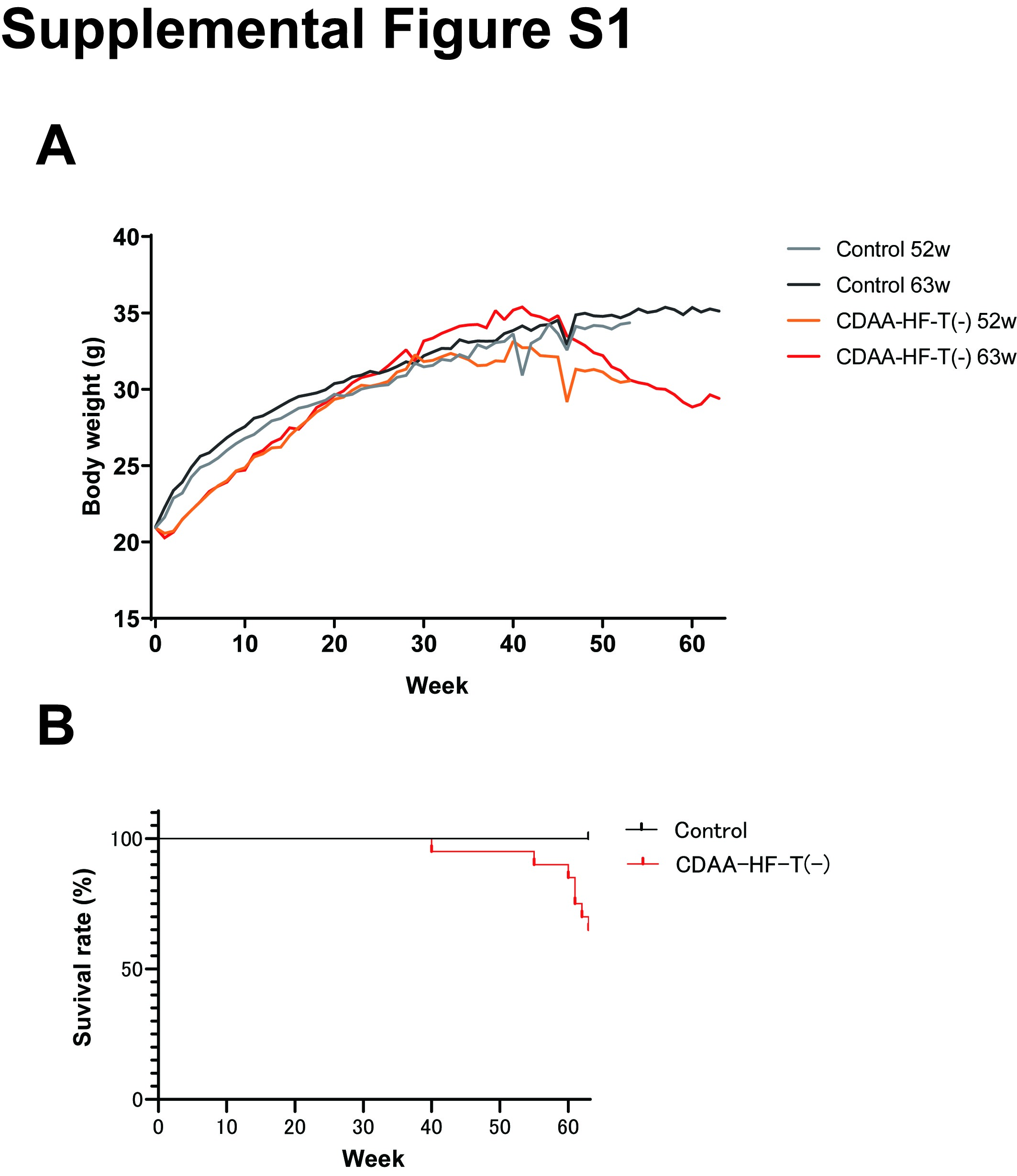

Supplement: S1 Fig — (A) Body weight changes and (B) survival rate of C57BL/6J mice fed with the control chow diet, CDAA-HF-T(−) 52 or 63 weeks. (TIF) [file pone.0287657.s001.tif]

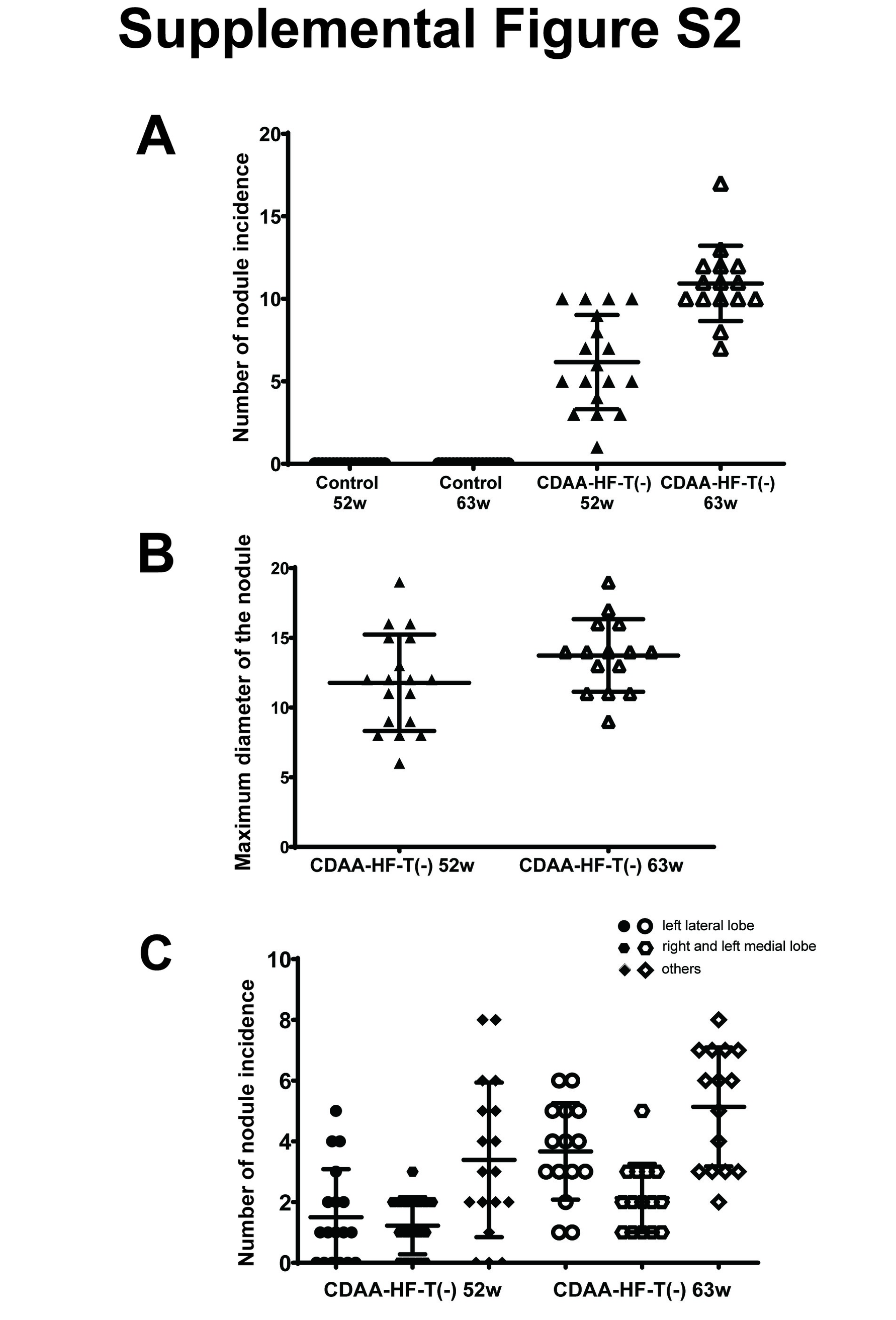

Supplement: S2 Fig — The number of the nodules in the left lateral lobes, medial lobes, and others in the liver of mice fed with CDAA-HF-T(−) for 52 and 63 weeks. (TIF) [file pone.0287657.s002.tif]

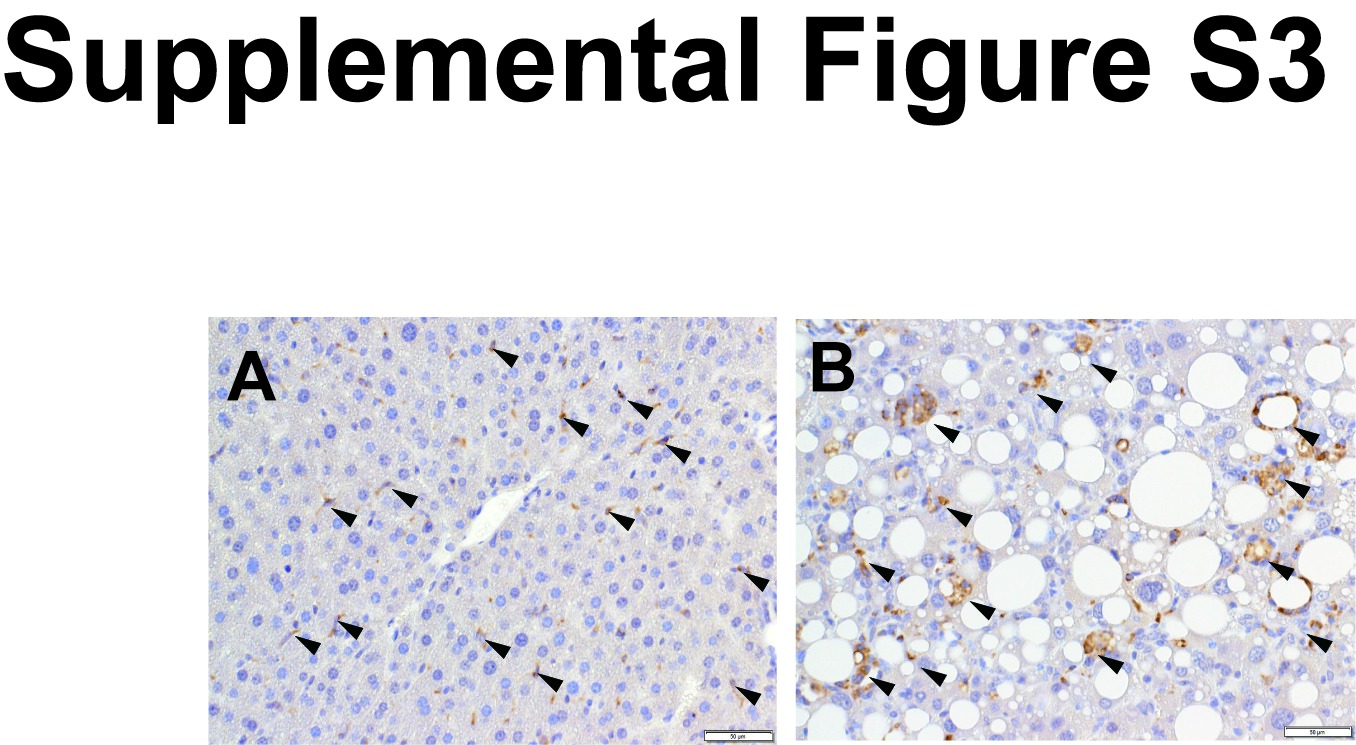

Supplement: S3 Fig — Representative immunohistochemical features for CD68 in the livers of mice fed with the control chow (A) and CDAA-HF-T(−) (B) for 13 weeks. Arrows indicate positive staining. (TIF) [file pone.0287657.s003.tif]

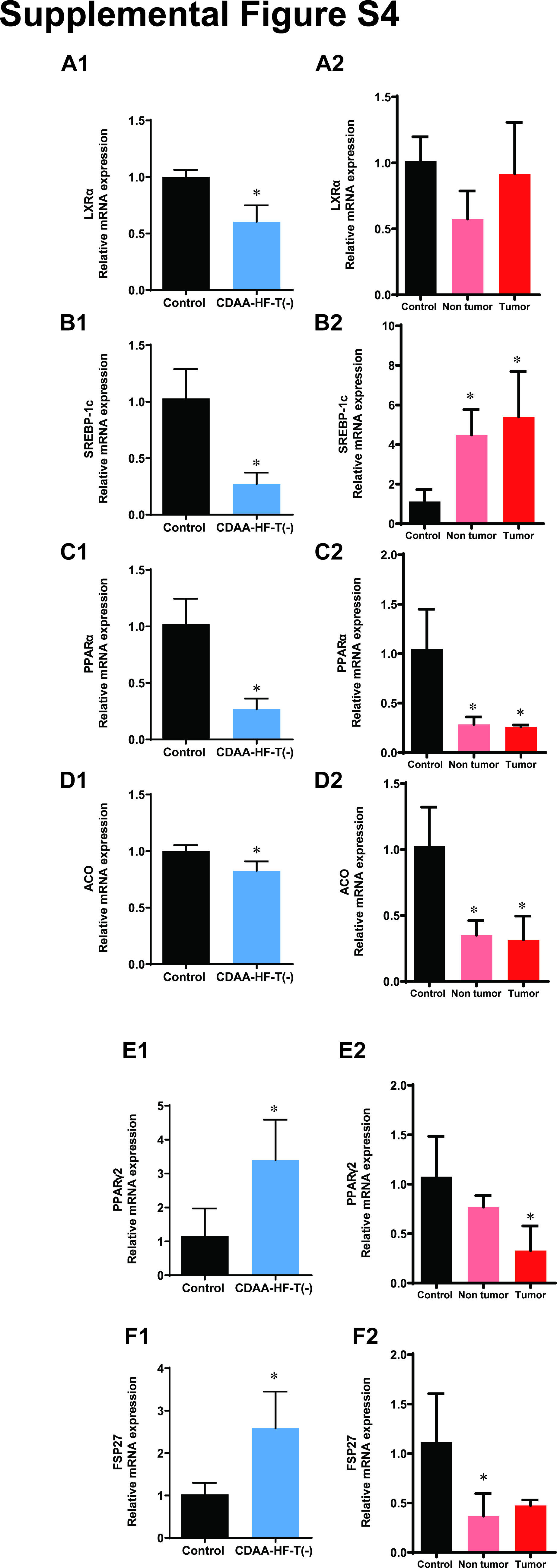

Supplement: S4 Fig — Quantitative real-time PCR of genes involved in LXRα (A), SREBP-1c (B), PPARα (C), and AOX (D) in the livers of mice fed with the control chow, CDAA-HF-T(−) for 13 weeks (A/B/C/D1) or 63 weeks (A/B/C/D2). *Significantly different from the control group value. Quantitative real-time PCR of genes involved in PPARγ2 (E) and FSP27 (F) in the livers of mice fed with the control chow, CDAA-HF-T(−) for 13 weeks (E/F1) or 63 weeks (E/F2). *Significantly different from the control group value. (TIF) [file pone.0287657.s004.tif]
