# Supplementary material for: Nonalcoholic steatohepatitis-associated hepatocarcinogenesis in mice fed a modified choline-deficient, methionine-lowered, L-amino acid-defined diet and the role of signal changes
Source: PLoS One. 2023 Aug 3;18(8):e0287657. doi: 10.1371/journal.pone.0287657 (PMC10399772; doi:10.1371/journal.pone.0287657)
Supplement: S1 Table — (DOCX) [file pone.0287657.s005.docx]

**S1 Table.** Sequence information of primers for the quantitative real-time PCR analysis

| **Gene** | **Forward** | **Reverse** |
| --- | --- | --- |
| **MIP-2** | **GATTCACCTCAAGAACATCCAGA** | **GGACACCTTTTAGCATCTTTTGG** |
| **PPARα** | **CCTCAGGGTACCACTACGGAGT** | **GCCGAATAGTTCGCCGAA** |
| **PPARγ2** | **AACTCTGGGAGATTCTCCTGTTGA** | **GAAGTGCTCATAGGCAGTGCAT** |
| **SREBP-1c** | **CGGCGCGGAAGCTGT** | **TGCAATCCATGGCTCCGT** |
| **LXRα** | **AGCAACAGTGTAACAGGCGCT** | **ACGATGGCCAGCTCAGTAAAGT** |
| **ACO** | **CGATCCAGACTTCCAACATGAG** | **CCATGGTGGCACTCTTCTTAACA** |
| **FSP27** | **GCTGAACCCTCAGGACTTTATT** | **CTTGTAGCAGTGCAGGTCATAG** |
| **36B4** | **CCTGAAGTGCTCGACATCACA** | **GCGCTTGTACCCATTGATGA** |
