# Supplementary material for: Nonalcoholic steatohepatitis-associated hepatocarcinogenesis in mice fed a modified choline-deficient, methionine-lowered, L-amino acid-defined diet and the role of signal changes
Source: PLoS One. 2023 Aug 3;18(8):e0287657. doi: 10.1371/journal.pone.0287657 (PMC10399772; doi:10.1371/journal.pone.0287657)
Supplement: S2 Table — (DOCX) [file pone.0287657.s006.docx]

**S2 Table.** Liver lobe weights at the ends of Weeks 52 and 63.

|  | **Control 52w** | **CDAA-HF-T(-) 52w** | **Control 63w** | **CDAA-HF-T(-) 63w** |
| --- | --- | --- | --- | --- |
| Left lateral lobe  (g) | 0.39±0.04 | 0.81±0.54 ^＊^ | 0.4±0.05 | 1.39±0.56 ^＊＃^ |
| Medial lobe  (g) | 0.36±0.03 | 0.64±0.37 | 0.37±0.04 | 0.73±0.64^＊^ |
| Others  (g) | 0.45±0.05 | 1.88±0.79^＊^ | 0.5±0.05 | 1.95±0.79^＊^ |
| Left lateral lobe/BW (%) | 1.13±0.11 | 2.39±1.73^＊^ | 1.15±0.14 | 4.54±1.78 ^＊＃^ |
| Medial lobe/BW (%) | 1.04±0.07 | 1.9±1.48 | 1.04±0.1 | 2.89±3.08^＊^ |

Values are means ± SDs.

*Significantly different from the control value.

^＃^Significantly different from the CDAA-HF-T(-) 52w value.
