# Supplementary material for: Nonalcoholic steatohepatitis-associated hepatocarcinogenesis in mice fed a modified choline-deficient, methionine-lowered, L-amino acid-defined diet and the role of signal changes
Source: PLoS One. 2023 Aug 3;18(8):e0287657. doi: 10.1371/journal.pone.0287657 (PMC10399772; doi:10.1371/journal.pone.0287657)
Supplement: S3 Table — (DOCX) [file pone.0287657.s007.docx]

**S3 Table.** Upregulated and downregulated genes in the upstream regulator, CDAA-HF-T(−)-*N* versus control

| **Upregulated** | **z-score** |
| --- | --- |
| Tumor necrosis factor | 10.126 |
| Interleukin-1β | 9.276 |
| Interferon gamma | 8.965 |
| Transforming growth factor β1 | 8.006 |
| Tretinoin | 7.42 |
| Transglutaminase 2 | 6.659 |
| Acute phase proteins | 6.283 |
| Cisplatin | 6.018 |
| Decitabine | 5.912 |
| Nitrofurantoin | 5.819 |
|  |  |
| **Downregulated** | **z-score** |
| Curcumin | -6.558 |
| SP600125 | -5.813 |
| Peroxisome proliferator-activated receptor α | -5.615 |
| Hepatocyte nuclear factor 4α | -5.252 |
| Sirtuin 1 | -5.145 |
| Polycystin 1 | -5.094 |
| Fenofibrate | -4.711 |
| Geldanamycin | -4.647 |
| 15-deoxy-delta-12,14 -PGJ 2 | -4.581 |
