# Supplementary material for: Nonalcoholic steatohepatitis-associated hepatocarcinogenesis in mice fed a modified choline-deficient, methionine-lowered, L-amino acid-defined diet and the role of signal changes
Source: PLoS One. 2023 Aug 3;18(8):e0287657. doi: 10.1371/journal.pone.0287657 (PMC10399772; doi:10.1371/journal.pone.0287657)
Supplement: S4 Table — (DOCX) [file pone.0287657.s008.docx]

**S4 Table.** Upregulated and downregulated genes in the upstream regulator, CDAA-HF-T(−)-T versus control

| **Upregulated** | **z-score** |
| --- | --- |
| Lipopolysaccharide | 8.449 |
| Tretinoin | 8.202 |
| Beta-estradiol | 7.423 |
| Tetradecanoylphorbol acetate | 7.114 |
| Interferon gamma | 7.078 |
| Angiotensinogen | 6.643 |
| Cisplatin | 6.347 |
| Transforming growth factor β1 | 6.168 |
| L-triiodothyronine | 5.958 |
| Tumor necrosis factor | 5.956 |
|  |  |
| **Downregulated** | **z-score** |
| Sirolimus | -4.511 |
| SWI/SNF related, matrix associated, actin dependent regulator of chromatin, subfamily a, member 5 | -4.43 |
| mir-155 | -4.262 |
| SMAD Family Member 7 | -4.199 |
| Wortmannin | -3.994 |
| Levodopa | -3.938 |
| Sirtuin 1 | -3.922 |
| Apolipoprotein A1 | -3.7 |
| Surfactant protein A1 | -3.681 |
