# Supplementary material for: Nonalcoholic steatohepatitis-associated hepatocarcinogenesis in mice fed a modified choline-deficient, methionine-lowered, L-amino acid-defined diet and the role of signal changes
Source: PLoS One. 2023 Aug 3;18(8):e0287657. doi: 10.1371/journal.pone.0287657 (PMC10399772; doi:10.1371/journal.pone.0287657)
Supplement: S5 Table — (DOCX) [file pone.0287657.s009.docx]

**S5 Table.** Upregulated and downregulated genes in the upstream regulator, CDAA-HF-T(−)-T versus CDAA-HF-T(−)-*N*

| **Upregulated** | **z-score** |
| --- | --- |
| Chorionic gonadotropin | 5.826 |
| Beta-estradiol | 5.536 |
| Hydrogen peroxide | 5.426 |
| Pirinixic acid | 5.246 |
| Transforming growth factor β1 | 4.679 |
| Neuregulin-1 | 4.644 |
| Nuclear factor erythroid 2 like 2 | 4.635 |
| Lipopolysaccharide | 4.489 |
| Jun proto-oncogene | 4.461 |
| Deferoxamine | 4.428 |
|  |  |
| **Downregulated** | **z-score** |
| PD98059 | -5.191 |
| SP600125 | -4.019 |
| Sirolimus | -4.016 |
| Aspirin | -3.644 |
| Actinomycin D | -3.442 |
| Imatinib | -3.34 |
| ZMPSTE24 | -3.228 |
| N-acetyl-L-cysteine | -2.937 |
| Tribbles pseudokinase 3 | -2.887 |
