# Supplementary material for: Nonalcoholic steatohepatitis-associated hepatocarcinogenesis in mice fed a modified choline-deficient, methionine-lowered, L-amino acid-defined diet and the role of signal changes
Source: PLoS One. 2023 Aug 3;18(8):e0287657. doi: 10.1371/journal.pone.0287657 (PMC10399772; doi:10.1371/journal.pone.0287657)
Supplement: S6 Table — (DOCX) [file pone.0287657.s010.docx]

**S6 Table.** Upregulated and downregulated genes in the canonical pathway, CDAA-HF-T(−) versus control, 13 weeks.

| **Upregulated** | **-log(p-value)** | **z-score** |
| --- | --- | --- |
| Dendritic Cell Maturation | 7.69 | 6.893 |
| Signaling by Rho Family GTPases | 5.57 | 6.364 |
| Neuroinflammation Signaling Pathway | 9.44 | 5.93 |
| Integrin Signaling | 6.64 | 5.742 |
| Tec Kinase Signaling | 5.86 | 5.741 |
| IL-8 Signaling | 7.28 | 5.582 |
| Colorectal Cancer Metastasis Signaling | 10.1 | 5.253 |
| PKCθ Signaling in T Lymphocytes | 7.4 | 5.196 |
| TREM1 Signaling | 9.02 | 5.191 |
| Fcγ Receptor-mediated Phagocytosis in Macrophages and Monocytes | 7.97 | 4.938 |
|  |  |  |
| **Downregulated** | **-log(p-value)** | **z-score** |
| RhoGDI Signaling | 3.97 | -4.814 |
| LXR/RXR Activation | 6.38 | -4.333 |
| PPAR Signaling | 1.25 | -3.8 |
| Antioxidant Action of Vitamin C | 4.94 | -3.157 |
| PTEN Signaling | 4.19 | -2.402 |
| Role of p14/p19ARF in Tumor Suppression | 3.33 | -1.897 |

| PPARα/RXRα Activation | 4.34 | -1.769 |
| --- | --- | --- |
| Inhibition of Matrix Metalloproteases | 3.36 | -1.5 |
| Cytotoxic T Lymphocyte-mediated Apoptosis of Target Cells | 0.834 | -1.414 |
| Amyotrophic Lateral Sclerosis Signaling | 3.87 | -1.372 |
