# Supplementary material for: Nonalcoholic steatohepatitis-associated hepatocarcinogenesis in mice fed a modified choline-deficient, methionine-lowered, L-amino acid-defined diet and the role of signal changes
Source: PLoS One. 2023 Aug 3;18(8):e0287657. doi: 10.1371/journal.pone.0287657 (PMC10399772; doi:10.1371/journal.pone.0287657)
Supplement: S7 Table — (DOCX) [file pone.0287657.s011.docx]

**S7 Table.** Upregulated and downregulated genes in the upstream regulator, CDAA-HF-T(−) versus control, 13 weeks.

| **Upregulated** | **z-score** |
| --- | --- |
| Lipopolysaccharide | 11.544 |
| Colony stimulating factor 2 | 10.177 |
| Tumor necrosis factor | 9.789 |
| Interferon gamma | 9.366 |
| Tretinoin | 8.799 |
| Poly rI:rC-RNA | 8.548 |
| Interferon alpha | 8.514 |
| Interleukin-1β | 8.376 |
| E. coli B4 lipopolysaccharide | 8.312 |
| Transforming growth factor β1 | 8.287 |
|  |  |
| **Downregulated** | **z-score** |
| SB203580 | -7.824 |
| Alpha catenin | -7.567 |
| tripartite motif containing 24 | -6.922 |
| Acyl-CoA oxidase 1 | -6.365 |
| mir-21 | -5.749 |
| LY294002 | -5.595 |
| Nuclear Receptor Subfamily 1 Group H | -5.517 |
| Suppressor of cytokine signaling 1 | -5.154 |
| Atypical chemokine receptor 2 | -5.099 |
| TSC complex subunit 2 | -5.079 |
